# Injuries and fatalities in Colombian mining emergencies (2005-2018): a retrospective ecological study

**DOI:** 10.47626/1679-4435-2022-799

**Published:** 2023-02-13

**Authors:** Gloria Catalina Gheorghe, Edgar F. Manrique-Hernández, Alvaro J. Idrovo

**Affiliations:** 1 Grupo Seguridad y Salvamento Minero, Agencia Nacional de Minería, Bogotá, Bogotá DC, Colombia; 2 Programa de Seguridad y Salud en el Trabajo, Escuela de Medicina y Ciencias de la Salud, Universidad del Rosario, Bogotá, Bogotá DC, Colombia; 3 Departamento de Salud Pública, Universidad Industrial de Santander, Bucaramanga, Santander, Colombia

**Keywords:** occupational health, mortality, mining, accidents, safety, saúde do trabalhador, mortalidade, mineração, acidentes, segurança

## Abstract

**Introduction:**

Mining injuries have decreased in a number of developed countries in recent decades. Although mining has become a very important sector of Colombia’s economy, no analyses of mining injuries and fatalities have been conducted.

**Objectives:**

This study describes the occurrence of mining emergencies in Colombia between 2005 and 2018 and their principal characteristics.

**Methods:**

This retrospective ecological study analyzed mining emergencies registered by the National Mining Agency between 2005 and 2018. The study described the place, event type, legal status, mine type, extracted mineral, and number of injuries and fatalities. Benford’s law was used to explore data quality.

**Results:**

A total of 1,235 emergencies occurred, with 751 injured workers and 1,364 fatalities. The majority of emergencies were from collapses, polluted air, and explosions, most of which occurred in coal (77.41%), gold (18.06%), and emerald (1.38%) mines. Many emergencies occurred in illegal mines (27.21%), most of which were for gold, construction materials, emeralds, and coal. Illegal mines had a higher relative proportion of injuries and fatalities than legal mines (p < 0.05). Mining disasters are likely to be underreported given that Benford’s Law was not satisfied.

**Conclusions:**

As mining increases in Colombia, so are mining emergencies, injuries, and fatalities. This is the first full description of mining emergencies in Colombia based on the few available data.

## Abstract (pt)

**Introdução:**

Durante as últimas décadas, vários países desenvolvidos relataram diminuição na ocorrência de acidentes em minas. A mineração é um setor muito importante da economia colombiana sem análises de acidentes e mortes em emergências em minas.

**Objetivos:**

Este estudo descreve a ocorrência e as principais características de emergências em minas na Colômbia entre 2005 e 2018.

**Métodos:**

Realizou-se estudo ecológico retrospectivo sobre emergências em minas registradas pela Agência Nacional de Mineração entre 2005 e 2018. O estudo descreveu o local da ocorrência, o tipo de evento, a situação jurídica e o tipo de mina, de mineral extraído e o número de acidentes e mortes. Utilizou-se a Lei de Benford para explorar a qualidade dos dados.

**Resultados:**

Ocorreu um total de 1.235 emergências, com 751 trabalhadores feridos e 1.364 mortos. A maioria das emergências foi decorrente de colapsos, contaminação do ar e explosões, principalmente em minas de carvão (77,41%), ouro (18,06%) e esmeralda (1,38%). Muitas emergências ocorreram em minas ilegais (27,21%), sobretudo de ouro, materiais de construção, esmeralda e carvão. As minas ilegais apresentavam maior proporção relativa de acidentes e mortes do que as minas legais (p < 0,05). É provável que os desastres em minas sejam subnotificados, uma vez que a Lei de Benford não foi cumprida.

**Conclusões:**

A Colômbia é um país com crescente atividade mineradora, e é crescente a ocorrência de emergências, acidentes e mortes em minas. Esta é a primeira descrição completa de emergências em minas na Colômbia com os poucos dados disponíveis.

## INTRODUCTION

Although injuries and fatalities are a frequent occurrence in the mining industry,^1^ mining injuries have decreased in recent decades in a number of countries in the European Union, as well as Canada and the United States.^2^ In contrast, mining injuries and fatalities in low- and middle-income countries have mostly occurred in informal high-hazard work in unsafe environments.^3^ In addition, historical analyses indicate that working conditions have improved in large open-pit mining, although its environmental sustainability has decreased.^4^ For these reasons, many recent studies on mining injuries and fatalities have been conducted in developing countries.^5-7^

Mining is an important activity in Latin America; oil, gas and various minerals are abundant. Between 2010 and 2015, there was a period of great bonanza in Bolivia, Colombia, Chile, Ecuador, Mexico, Peru and Venezuela. Despite this growth in production and economic profitability, much mining occurs in regions with high levels of poverty and social vulnerability.^8^ In this context, mining injuries and fatalities primarily affect disadvantaged individuals. Unfortunately, few studies have rigorously explored the occurrence of injuries and fatalities in mines in Latin America. Thus, secrecy and oblivion surround this subject of great importance for occupational health and safety.

Mining has become a key industry in Colombia in recent decades, surpassing agriculture and other formerly dominant industries.^9^ The largest operations involve the extraction of coal, ferronickel, and gold, followed by building materials, salt, silver, and platinum.^10^ Despite the growing importance of mining to Colombia’s economy, occupational health and environmental studies of the mining sector are scarce. In 2018, roughly 12% of miners were injured, and the fatality rate was 73 per 100,000 workers.^11^ Mining emergencies are complex events involving different injury and fatality rates; understanding their characteristics could help prevent their occurrence. Since mining emergencies are group events, the results can complement data on individual worker injuries and allow identification of preventable determinants.

The most notorious Latin American mining emergencies in recent memory were: the 2006 Pasta de Concho disaster, in which an explosion due to accumulated gases caused a coal mine to collapse, resulting in 65 fatalities and 13 injuries in San Juan de Sabinas, Coahuila, Mexico; the 2007 collapse of a gold mine in Suárez, Cauca, Colombia, which caused 24 fatalities and more than 30 injuries; the 2010 collapse of the San José copper mine in Atacama, Chile, which trapped 33 miners underground for 69 days; the 2010 explosion in a gold mine in Amagá, Antioquia, Colombia, which caused 73 fatalities; and the 2015 Mariana dam disaster in Minas Gerais, Brazil, which caused 19 fatalities and more than 16 injuries. However, this list of mining emergencies alone does not indicate the complex nature of mining safety issues in the region.^12,13^ Given this context, the objective of the present study was to describe the occurrence of injuries and fatalities caused by mining emergencies in Colombia from 2005 to 2018.

## METHODS

A retrospective ecological study was performed with mining emergencies in Colombia as the unit of analysis.^14^ The study reviewed emergency statistics recorded between 2005 and 2018 by the *Agencia Nacional Minera* (National Mining Agency), which is the mining authority in Colombia. Its functions include granting mining contracts to private entities, managing land registries, monitoring compliance with contractual obligations, including technical, legal, and financial obligations (royalty payments and other financial compensations), mining health and safety, and coordinating the National Mining Rescue System. National Mining Agency reports on mining emergencies are public, although the name and identification of involved workers are not included in the databases; however, at its discretion, the agency may provide such data through official requests detailing the usage of the data. According to Colombian law, studies using anonymous public data do not require approval by an ethics committee.

The variables included in the analysis were the date of the emergency, the extracted mineral, the place (province, municipality, and town), mine type (open pit or underground), legal status (legal or illegal mine), event type (fall from a height, collapse, electrical, mechanical, explosion, slope instability, fire, flood, polluted air, or events involving heavy machinery), and the number of injuries and deaths.

The statistical methods consisted of using percentages to describe the categorical variables, while measures of central tendency and dispersion were used to describe quantitative variables, based on evaluating the distribution with the Shapiro-Wilk test. The Mann-Whitney test was used to explore possible differences in the occurrence of injuries and fatalities in legal vs illegal mines. In addition, the quality of the data was evaluated by applying Benford’s Law (for first and second digits) to the injury and fatality data. In the analysis of first digits, data with zeroes were excluded.^15,16^ The digits macro-developed by Ben Jann (ETH Zurich) was used for that purpose, and Pearson’s x^2^ and log likelihood ratio were used to evaluate the goodness-of-fit of the distributions. Stata 14 (Stata Corporation, College Station, TX, USA) was used for these analyses.

## RESULTS

**Figure 1:**
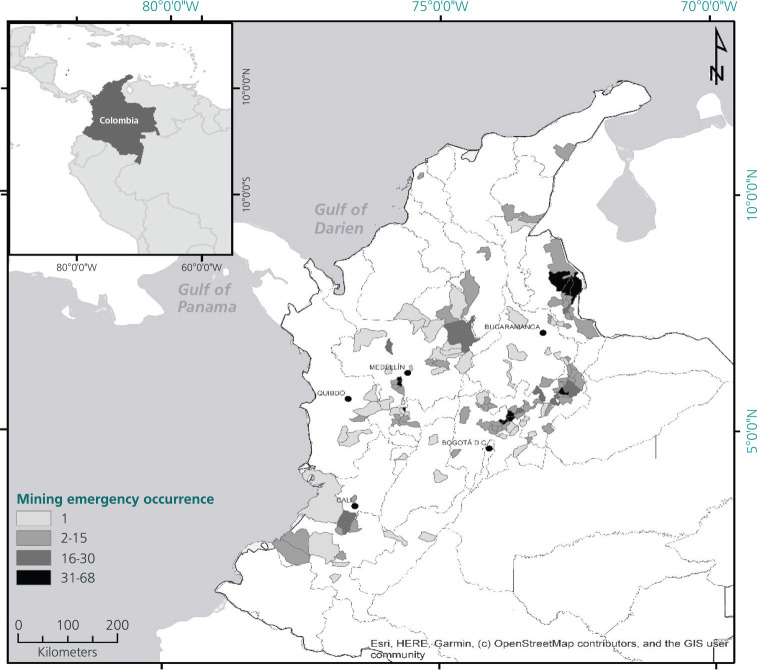
Map of sites with mining emergencies in Colombia (2005-2018).

**Figure 2:**
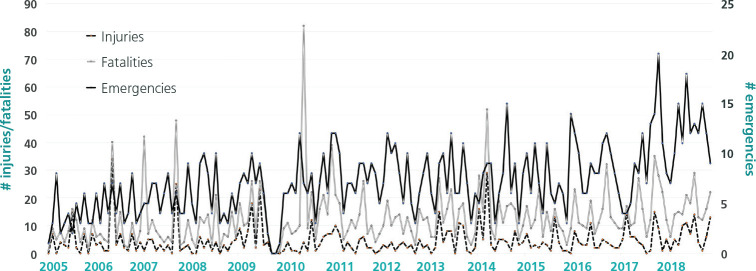
Temporal trend of injuries and fatalities in mining emergencies in Colombia (2005-2018).

The results present analysis of the time and place variables first, followed by worker-related variables. A total of 1,235 mining emergencies occurred in Colombia between 2005 and 2018, with 751 injured workers and 1,364 fatalities. Thus, a mining worker was injured approximately every 6.8 days, and a miner died from a mining emergency every 3.75 days. Figure 1 shows the sites where the mining emergencies occurred. As can be seen, the municipalities with the most emergencies were Cucunuba (n = 68), Lenguazaque (n = 61), and Guacheta (n = 52) in Cundinamarca; Marmato (n = 55) in Caldas; Amagá (n = 49) and Angelópolis (n = 40) in Antioquia; and Tasco (n = 41) in Boyacá. Note the high concentration of mining emergencies in regions with a sustained tradition of underground mining. Figure 2 presents the occurrence of mining emergencies by month, showing large monthly variability in the number of emergencies (median 7, min 0, max 20), injuries (median 3, min 0, max 34), and fatalities (median 7, min 0, max 78). There is no evidence that the rate of mining emergencies changed over time.

Table 1 summarizes the main characteristics of the mining emergencies in Colombia between 2005 and 2018, the majority of which involved few injured workers (97.81%). Fatalities were similarly distributed, with the majority of the emergencies involving fewer than five deaths (97.49%). The trend towards few injuries and fatalities was expected regarding this type of disaster. Most emergencies were due to collapses, polluted air, and explosions, which together represented 59.59% of the total. This can be explained by the fact that most of the affected mines are underground. The higher number of emergencies occurred on Wednesdays and the lowest on Saturdays and Sundays, two days that typically are not workdays in Colombia. The most significant mine types were coal, gold, and emerald, which together represented 96.85% of the total. This finding reiterates that the underground mines were most affected.

**Table 1:** Characteristics of mining emergencies in Colombia (2005-2018)

Variable	n	%	95%CI
Injured workers			
0	919	74.41	71.88-76.83
1	175	14.17	12.27-16.24
2	67	5.43	4.23-6.84
3	32	2.59	1.78-3.64
4	15	1.21	0.68-2.00
5-28	27	2.19	1.45-3.17
Fatalities			
0	409	33.12	30.49-35.82
1	639	51.74	48.91-54.56
2	110	8.91	7.38-10.64
3	36	2.91	2.05-4.01
4	10	0.81	0.39-1.48
5-73	31	2.51	1.71-3.54
Type of emergency			
Fall from height	46	3.72	2.74-4.94
Collapse	412	33.36	30.73-36.07
Electrical	34	2.75	1.91-3.83
Mechanical	119	9.64	8.05-11.42
Explosion	155	12.55	10.75-14.23
Slope instability	49	3.97	2.95-5.21
Fire	98	7.94	6.49-9.59
Flood	27	2.19	1.45-3.17
Polluted air	169	13.68	11.82-15.73
Heavy machinery-related	2	0.16	0.02-0.58
Other	50	4.05	3.02-5.30
No data	74	5.99	4.73-7.46
Day of week			
Sunday	42	3.40	2.46-4.57
Monday	170	13.77	11.89-15.81
Tuesday	222	17.98	15.87-20.23
Wednesday	239	19.35	17.18-21.67
Thursday	220	17.81	15.72-20.06
Friday	212	17.17	15.10-19.39
Saturday	99	8.02	6.56-9.67
No data	31	2.51	1.71-3.54
Mineral			
Coal	956	77.41	74.97-79.71
Gold	223	18.06	15.95-20.32
Emerald	17	1.38	0.80-2.19
Phosphoric rock	6	0.49	0.17-1.05
Limestone	5	0.40	0.13-0.94
Sand	5	0.40	0.13-0.94
Construction materials	4	0.32	0.08-0.83
Hydrocarbon	2	0.16	0.02-0.58
Sulfur	2	0.16	0.02-0.58
Gravel	1	0.08	0.00-0.45
Dragline spoil material	1	0.08	0.00-0.45
Copper	1	0.08	0.00-0.45
Salt	1	0.08	0.00-0.45
No data	11	0.89	0.45-1.59
Legal status			
Legal	828	67.04	64.34-9.66
Illegal	336	27.21	24.74 -29.78
No data	71	5.75	4.51-7.20

95%CI = 95% confidence interval.

The majority of the mines in which emergencies occurred were legal. Nevertheless, it is of interest that 56.50% of the gold mines, 52.94% of the construction material mines, 33.33% of the emerald mines, and 20.71% of the coal mines were not legal. These findings show that a great deal of mining activity is carried out beyond government control, despite its high risk. Occupational health and safety has not reached all Colombian mines. The emergencies in illegal mines were due to slope instability (57.14%), flooding (48.15%), polluted air (34.32%), and collapses (31.80%). The relative frequency of injuries and fatalities was greater at illegal mines than at legal mines (p < 0.05, Mann-Whitney test). This demonstrates that mining is safer when regulations are followed, including the presence of occupational health and safety personnel.

Table 2 shows the emergency types of according to extracted mineral. Most notably, all types of emergencies were more frequent in coal and gold mines. Collapses were most frequent type overall (33.36%), followed by polluted air (ie, oxygen-deficient atmosphere due to displacement by methane and carbon monoxide) (13.68%), explosions (most of which were associated with methane and/or dust in coal mines) (12.55%), mechanical failure (9.64%), and fires (7.94%). Unstable slopes were important in alluvial gold mining, especially illegal extraction. This detailed description allows us to understand the particular characteristics of each type of mine, as well as possible interventions for improving the health and safety of workers.

**Table 2:** Occurrence of mining emergencies according to mineral type, Colombia (2005-2018)

Mineral	Type of emergency												
	Fall from height	Collapse	Electrical	Mechanical	Explosion	Slope instability	Fire	Flood	Polluted air	Relate heavy machinery	Other	No data	Total
Coal	31	321	27	105	121	5	93	21	139	2	39	52	956
Gold	13	79	7	11	32	30	3	6	25		7	10	223
Emerald		4			1	1							6
Phosphoric rock	1			1	1	1	1						5
Limestone		1				2					1		4
Sand						1					1		2
Construction materials	1	7				4			4		1		17
Hydrocarbon				1					1				2
Sulfur						4						1	5
Gravel						1							1
Dragline spoil material				1									1
Copper												1	1
Salt							1				1		2
No data												11	11
Total	46	412	34	119	155	49	98	27	169	2	50	74	1,235

Finally, Table 3 presents the data quality assessment, which shows a deviation from Benford’s law of first digits. Assessment of the second digits resulted in a similar finding (data not shown), which suggests the number of workers affected by mining emergencies has been an underreported.

**Table 3:** Expected and observed frequencies of digits based on Benford’s law for the occurrence of injuries and fatalities in Colombian mining emergencies (2005-2018)^*^

Digit	Expected (%)	Observed			
		Injuries (%)	p-value	Fatalities (%)	p-value
1	30.103	58.228	< 0.0001	77.966	< 0.0001
2	17.609	22.152	0.3840	13.559	0.0019
3	12.494	10.127	0.2332	4.479	< 0.0001
4	9.691	4.747	0.0016	1.211	< 0.0001
5	7.918	1.266	< 0.0001	0.847	< 0.0001
6	6.695	1.266	< 0.0001	0.605	< 0.0001
7	5.799	1.582	0.0004	0.605	< 0.0001
8	5.115	0.316	< 0.0001	0.484	< 0.0001
9	4.576	0.316	< 0.0001	0.242	< 0.0001
Pearson’s X^2^		p < 0.0001		p < 0.0001	
Log likelihood ratio		p < 0.0001		p < 0.0001	

* Mining emergencies with zero occurrences were excluded.

## DISCUSSION

Our findings suggest a high occurrence of mining emergencies in Colombia, with no clear decreasing trend over time. This is related to the increasing number of mines in the country as a result of the mining-energy boom over the past two decades.^9^ At first glance, the fact that there were more fatalities than injuries may seem contradictory. One explanation is that the data corresponded exclusively to mining emergencies; our study did not include occupational injuries unrelated to mining emergencies. The occurrence of more fatalities than injuries is frequent in mining emergencies, as seen in the best-known cases in Latin America. Moreover, the deviation from Benford’s distribution suggests the underreporting of cases.^17^

Based on analyses conducted in other countries, this type of result cannot be separated from other occupational health and safety findings in Colombian mines. The concentration of suspended particles in the air is a good indicator of industrial safety in coal mines. In open-pit mines, which by definition have a lower concentration than underground mines, the findings indicate wide variability in Colombia, from complying with international standards to very high values, such as average annual PM_10_ concentrations over 70 µg/m^3^.^18^ In underground coal mining, an association has been reported between air pollution levels and pneumoconiosis, which has reached up to 35.9% for miners with more than 10 years’ experience.^19^ Spirometric signs, symptoms, and findings also suggest respiratory problems.^20^ The moral, legal, and economic costs associated with coal mining are greater than the economic benefits of extracting and exporting the mineral.^21^ In general, when there is evidence of high concentrations of contaminants, the risk of explosion is expected to be higher.

Gold mining has been related to serious environmental and social problems, including human health, especially due to the extensive use of mercury in many regions of the country.^22^ Various effects have been seen in diverse populations of workers and the general community.^23^ Regarding underground gold mining, a lack of control over mercury use has led to excessively high levels in urban air, which have been reported as the highest worldwide, with up to 1 million ng/m^3^ inside gold shops.^24^ Emerald mining has been strongly linked with violence in Colombia,^25^ which has impeded the study of safety and health conditions at mining sites. Violence has also been associated with illegal gold mining.^26^ Both gold and emerald mines are good examples of the need for appropriate understanding and management of the social context. In Colombia, these mines are frequently associated with various types of violence, including that of illegal armed groups.

Certain limitations should be considered regarding our findings. Although we used official records from the institution responsible for addressing mining emergencies in Colombia, the data quality assessment and the fact that there were more fatalities than injuries strongly indicate underreporting, as was also observed during the H1N1 influenza epidemic.^27^ The evidence presented in this study suggests that the majority of unreported emergencies would have been related to illegal mining, the extraction of gold, or problems with slope stability. Based on the findings of this study, as well as a more complete analysis of mining emergencies, it will be possible to improve the data input for further research into their causes.

## CONCLUSIONS

The occurrence of mining emergencies in Colombia is high, and they affect a significant number of workers. Without a doubt, this country has one of the highest rates of mining emergencies, which is a consequence of the difficulties of improving safety in mines and their contexts. A joint effort is needed among governmental agencies, mine owners, companies that provide liability insurance, and academic institutions to bring about cultural changes that prevent mining accidents and promote legal mining and compliance with occupational health and safety laws, as well as environmental protection. Mining rescue will continue to pose a challenge as long as economies are based on mineral extraction. It is highly advisable for the Colombian mining authority to design and manage a system that enables it to consolidate data on all serious and deadly accidents, as well as the causality analysis results of all conducted investigations. Future efforts should prioritize training and research to control the main mining risks, including underground mine ventilation, geomechanics, hydrogeology, control of explosions associated with methane and coal dust, and the evaluation of risks and hazards. Additionally, government social programs are required in the regions where mining is carried out to better address the social determinants that impact mining safety.
